# A qualitative exploration of patients’ perception regarding the comprehensive dental services availed at a primary health center

**DOI:** 10.12688/f1000research.146781.2

**Published:** 2024-05-28

**Authors:** Shushma Rao B, Ramya Shenoy, Parul Dasson Bajaj, Ashwini Rao, Mithun Pai, Praveen Jodalli, Avinash BR, Aparna KS, Navya Shinaj, Shagufta Musheer

**Affiliations:** 1 Department of Public Health Dentistry, Manipal College of Dental Sciences Mangalore, Manipal Academy of Higher Education, Manipal, Karnataka, 576104, India

**Keywords:** Comprehensive oral health care, patient perception, patient satisfaction, primary health center, public-private partnerships, health and wellbeing

## Abstract

**Introduction:**

Comprehensive oral care is a service centered around the patient, and individuals who need it the most often face limited access. Patient perception acts as a guide for enhancing quality, ensuring patients’ future intent to utilize the services and facilitating recommendations to others. The present study aimed to assess the patients’ perception of comprehensive dental services availed at a Primary Health Center (PHC).

**Methods:**

This qualitative study was based on a phenomenological interpretive approach, and judgment sampling method was employed. A validated interview guide, developed from relevant literature was employed in the local language to conduct interviews among adults visiting the PHC, gathering their views regarding the services provided. The interviews were audio recorded on a digital voice recorder, and files were password protected. Content saturation guided the determination of the final number of participants interviewed. After translating and transcribing the interviews, thematic analysis and coding were performed using ATLAS. ti 23 for Windows.

**Results:**

A total of 12 participants were included in the study, following data saturation. Among them, there were 8(66.7%) female and 4(33.3%) male participants. Ten overarching main themes were discerned through the assigned codes, including positive views, neutral views, negative views, previous dental clinics visited, previous experience with dental treatment, treatments sought at the center, referrals, source of information about the dental center, subsequent visits and suggestions for improvement.

**Conclusions:**

The findings of this study revealed a positive patient perception of the comprehensive dental services offered at the PHC. Through insightful interviews, various strengths, and areas for improvement regarding the center and care provision were identified. These insights provide valuable suggestions that can be applied to elevate the utilization of dental services, ensuring continuous improvement in patient care.

## Introduction

The Dawson report first mentioned the term ‘Primary Health Care Center’ around 1920 in the United Kingdom. ^1^ Primary care is defined as “the provision of integrated, accessible health care services by clinicians who are accountable for addressing a large majority of personal health care needs, developing a sustained partnership with patients, and practicing in the context of family and community.” ^2^ Although the World Health Organization (WHO) has recognized the importance of primary care in health service systems, there is dearth of primary care physicians compared to specialist physicians. ^1^^,^^3^ A similar shortage is observed in Sub-Saharan Africa, where factors such as inadequate financing and unreliable expenditure on medicines add to the challenges of providing primary health care. ^4^^–^^9^

In India, Primary Health Centers (PHCs) and its outreach teams constitute the “backbone” of the rural health care system with strong referral mechanisms connecting them to speciality care. ^10^ However, in developing countries, primary health centers face challenges in delivering oral health care, necessitating programs to enhance community outreach, meet basic health requirements, and facilitate proper patient referrals. ^11^ The government of India launched the National Oral Health Programme to provide comprehensive oral health care in response to the oral health conditions in the nation. This initiative aims to improve the determinants of oral health, integrating oral health promotion and preventive services with the general health care system, and encouraging the promotion of public-private partnerships (PPP) as a means of improving oral health. ^12^ The provision of comprehensive oral care is a patient-centered service ^13^ that encompasses various dental treatments, including examinations, consultations, prophylaxis, and restorations. ^14^ However, it is typically observed that while the population in urban areas has limitless access to these services, those in greater need of them have restricted access. ^15^

In an effort to address the needs of underserved populations, community health services step in to offer free dental services, bridging the gap in access to oral care. ^16^ Despite existing barriers such as challenges related to transportation and caregiver attitudes, ^17^ literature suggests that these programs contribute to a reduction in carious teeth, particularly in children. ^13^ To optimize services, co-locating dental clinics within medical health centers proves beneficial, streamlining the referral process and providing patients with convenient access to both medical and dental care. ^18^ The sustainability of such services can be enhanced by gaining an in-depth understanding of the perceptions of the patients regarding the services availed. Therefore, a qualitative approach is apt for comprehensively understanding the influences and contexts that affect the decision-making processes of individuals regarding their oral health care needs.

Patient satisfaction, as evidenced in various studies, plays a crucial role in ensuring compliance, improving clinical outcomes, and increasing service utilization. ^19^ Patient satisfaction is affected by patient perception, ^20^ which is based on their dental experiences and a certain level of expectation when they avail dental services from a primary health center at a reasonable to no cost. ^21^ Furthermore, it serves as a roadmap for enhancing quality, ^22^ ensuring that patients not only have the intention to use the services in the future themselves but also to recommend them to others. ^23^^,^^24^ Hence, the present study was carried out to assess the patients’ perception towards comprehensive dental services availed at a Primary Health Center (PHC) located in South India.

## Methods

The present qualitative research, which explores the perceptions of patients receiving comprehensive oral health care, has been reported in accordance with the COREQ (Consolidated criteria for Reporting Qualitative research) Checklist. ^25^ The materials used in this study (questionnaire, participant information sheet, interview guide, sample consent form and filled COREQ checklist for the present study) are available as extended data. ^26^

### Ethical considerations

Permission of Head of the Institution was obtained. Ethical approval was obtained before commencement of the study (Protocol No. 22105 dated 3 ^rd^ January, 2023) from the Institutional Ethics Committee of Manipal College of Dental Sciences, Mangalore. Written informed consent was taken and participation was completely voluntary. Confidentiality of the participants was maintained.

### Study design

This qualitative study was based on phenomenological interpretive approach. It goes beyond mere description to uncover the meanings, where the researcher attempts to interpret patients’ perception based on their judgements. ^27^

### Study setting

The study was conducted at a Primary Health Center situated in Southern India. The dental care services at this center are delivered through a well-established public-private partnership formed between the Primary Health Center and a private dental college. This facility serves a population of 63,000 individuals and encompasses nine sub-centers.

### Participant selection

The study participants were selected from among the adult patients (aged 18 years and above) seeking dental health services at the PHC, particularly those who had follow up visits for various dental treatments. This selection was based on judgement sampling, a non-probability sampling method that enables maximum variation in the sample. This approach enhances the acquisition of more significant insights into the phenomenon from various perspectives, providing flexibility in capturing a broad range of viewpoints. Participants were given details about the study’s objectives and the rationale for its execution. An oral as well as written consent was obtained from each participant before the interviews. ^26^

### Sample size

Sample size is estimated to be maximum of 25 people as per literature. ^28^ The final number of participants depended on content saturation, which was inferred when the codes and themes generated after each interview became repetitive. ^27^

### Data collection

The interviews were conducted by a female dental post-graduate resident trained in qualitative research methods, fluent in the local language and who regularly attended postings at the PHC (SR). The interview guide ^26^ was developed based on both existing literature ^9^^,^^18^^–^^21^ and insights from two researchers who have been actively engaged in the program since its commencement. These researchers had firsthand experience in delivering clinical care to the local population and were well-versed in conducting interviews. The interview guide consisted of open-ended questions to gain a deeper understanding of participant’s perceptions and was translated to the local language. Face and content validity was performed by three subject experts and the interview guide was pilot tested on two adult patients who were not included in the study for clarity and comprehension. A separate room was identified in the PHC to carry out the face-to-face in-depth interviews, conducted in the local language with interview times ranging from 5–14 minutes. The interviews were audio recorded on a digital voice recorder by a keynote taker and the files obtained were stored in password protected computers. The consent of the participants was obtained both orally and in writing prior to the interview, ensuring their anonymity to make them comfortable in answering the questions honestly, thereby addressing social desirability bias. At the end of each session, the investigator summarized the key points derived from the discussion. The demographic details of the participants like age, gender and area of residence were also collected. There were no repeat interviews carried out.

### Data analysis

The interviews were translated in English by one investigator (SR) from the local language and transcribed verbatim for subsequent data analysis, along with deidentification of the transcripts. The translated transcripts were read and reread by two investigators (SR & RS) independently for ensuring accuracy in the transcripts, with both investigators being proficient in the local language as well as in English. Any disagreements were jointly discussed by the two investigators while revisiting the audio recordings. The investigators read the interview transcripts several times to thoroughly understand the content and grasp the context of the responses. After gaining a deep understanding, the investigators highlighted important keywords in the text. This was followed by identifying the initial codes in the interview transcripts to organize the data and identify any emerging patterns. After an extensive discussion with a third researcher (PDB), the initial codes were further refined to arrive at a final list of codes, and subsequently categories, subthemes and main themes from the entire dataset were identified and compiled. Coding and thematic analysis were conducted using ATLAS.ti software version 23 for Windows. ^29^ Each study participant was consistently referred to by their assigned codes throughout the data analysis by all members of the research team. To maintain confidentiality, all data, including audio recordings and transcripts, were securely stored, and preserved in password-protected computers accessible solely to the research members involved in the study.

## Results

A total of 12 participants were interviewed in this study with a mean age of 49.42 (±15.9) years. Among these, 8 (66.7%) were female, and 4 (33.3%) were male with the majority of participants lived in close proximity to the PHC.

The qualitative examination of the interviews resulted in the identification of three primary themes: pre-visit perspectives, visit related perspectives, and post-visit perspectives. Each theme was subsequently subdivided into two sub-themes, with further categories emerging from the respective codes. A comprehensive overview of these themes, sub-themes, categories, and codes is presented in Table 1.

**Table 1.** A visual representation outlining themes derived from the analysis of the interviews.

Theme	Sub-theme	Categories	Codes
**Pre-visit perspectives**	**Past dental experience**	Previous dental clinics visited	Private Dental College
			Private Dental Clinic
			Camp organized by a private dental college
		Past dental treatment and related issues	High cost of dental treatment
			Never visited a dental clinic/Rarely seeks treatment
			Previous treatment sought: cleaning of teeth, dentures and root canals.
	**Source of information about the PHC**	-	Government Hospital
			People who have visited the remote center /Local People/ Passerby/ Workplace
			Organized group/Social Worker
			Relatives/Neighbours
			Visiting PHC for medical treatment
			To avail free treatment
**Visit related perspectives**	**Treatments sought at the PHC**	Conservative dentistry & Endodontics	Broken tooth
			Tooth decay
			Tooth filling
			Toothache
			Root canal
		Periodontology	Cleaning Teeth
		Oral Surgery	Tooth Extraction
		Prosthodontics	Denture
		Pediatric dentistry	Visited for child's treatment
	**Feedback about the center**	Positive	Better oral health post treatment
			Free dental treatment
			Happy with behavior of staff/ No discrimination
			Satisfied with comprehensive care at the center
			Well located center
			Accessible dental treatment
			No waiting time
		Neutral	Skeptical about denture
			Waiting time acceptable
		Negative	Less dental chairs at center
			Difficulty to reach
			Waiting time
**Post-visit perspectives**	**Endorsement of PHC for dental treatment**	Recommended the PHC for dental care	For free treatment
			Recommended to others
		Reason for subsequent visits	On their own to seek treatment
			Revisited for appointments
	**Suggestions to improve the dental care at the center**	Cost related	Reduce rates for fixed dentures
			Provide crowns for free
		Service and Infrastructure related	Provide access to oral health care for adults and children in one location
			Provision of Crowns/fixed dentures treatment at the PHC
			Availability of drinking water
			Install more dental chairs
			Provision of shade in the waiting area
		Personnel related	Increase the number of dentists in the center
			More experienced and specialist dentists

**Figure 1.**
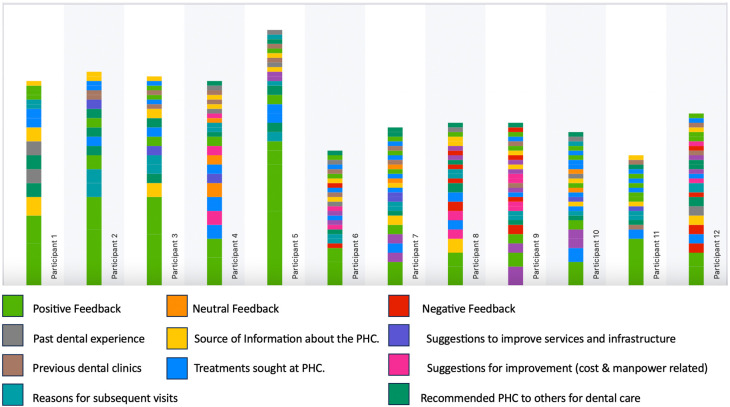
The color-coded chart depicting the occurrence of codes in each participant interview.

Each category was assigned a distinct color to facilitate easy identification of the codes in the transcribed interviews. The color-coded chart, depicting the occurrence of codes in each participant interview, is presented in Figure 1 and aids in recognizing the frequency of each code in every participant interview. Each theme, along with illustrative quotes, are detailed below.

### Pre-visit perspectives

This main theme was further divided into two subthemes including past dental experience and source of information about the dental care at PHC. The majority of participants (n = 6) had sought dental care at private dental clinics, while others visited dental camps or private dental colleges for treatment. One participant (P5) provided a detailed account of visiting multiple dental clinics for treatment, expressing dissatisfaction, as evident in the following quotation:

*P5: “… there was an old aged doctor, nearby only. He had own clinic below his home. He is a good doctor. Cheap and best. When I went to him, I had problem of severe sensitive teeth and pain too, so what he did every time he used to keep clove oil and send. Then I used to have gum problem too. He gave ointment to apply for one year. Then what I did was I went to another dentist. Then these gum treatment people couldn’t correct my problem. Then I went to another place near this nataraj theatre. They were THE BEST. Then they gave me tablet becosules. Everything got cured in one month. Then I had to get set done from him for my grandchild’s wedding…”*

When discussing past dental experiences, participants had sought various treatments, including scaling, prosthetic rehabilitation, and root canal treatment. Interestingly, two participants (P1 and P10) had never visited a dental clinic or undergone any kind of dental treatment, as indicated in the following excerpt:

*P10: “No. This is my first time I came for my dental treatment.”*

While two participants (P8 and P12) cited the high cost of dental treatment as a reason for not seeking dental services earlier, this ultimately motivated them to seek free dental care at the PHC, as elaborated in the following excerpt:

*P12: “I am a beedi worker, if I put in all my money for dental treatment then what will be left. Some take 600 rupees for extracting a tooth. I have put so much money for treatment in honnakatte; 1500, 4000 and 3000 rupees. And no benefit also. I got three dentures done but I can’t wear them. I have spent a lot.”*

The participants obtained information about the center from various sources, such as acquaintances, local residents, social workers, and individuals seeking medical treatment at the PHC. Three female participants (P7, P8, and P12) mentioned that they visited the center upon learning about the availability of free treatment, as depicted in the following excerpts:

*P8: “For root canal and for crown. I was told it is free here. So, I came.”*

*P12: “One of my relative said that in Surathkal they do good dental treatment and why to pay and go elsewhere. So, I came here. Two three people have come there from our area.”*

### Visit related perspectives

Participants sought dental care at the PHC, encompassing services like scaling, restorations, root canals, prosthetics, and tooth extraction, as evidenced by the following quotes:

*P6: “I had lot of toothache. They told root canal needs to be done. For that I came here.”*

*P5: “I came here only for teeth set. I had no teeth at all. That’s why I came here for teeth set. Then in 5 sittings I got my denture. Both up and down.”*

Predominantly, participants provided positive feedback about various aspects of the primary health center, covering aspects such as the availability of free treatment and the comprehensiveness of care, as evidenced by the excerpts:

*P6: “Many people wanted such a center to open. Like I had shown my teeth in another place. They told the cost for treatment was 12000 rupees. But here since root canal is done for free it is helpful to many people. Its easy to come and go too.”*

*P7: “My opinion is good only. If everything is available at one place then it is easy right? It is helpful for us. It saves our time and yours.”*

*P3: “Improvement is not needed. Just keep continuing like this only then it is good. I have told someone that we can come here, better than going to private they can get treatment here. They do nicely here.”*

*P6: “Yes. Earlier when that pain used to start, I would find it difficult even to talk. Now it is better.”*

A few participants also appreciated the behavior of the staff, specifically noting the absence of discrimination at the center, as seen in the following excerpt:

*P5: “…But that first one I had to pay 20000 and this was for free. When we compare the two you all are giving great service to society.… That too without considering if person is poor or able, no difference between BPL and APL.”*

Negative feedback focused around long waiting time and difficulty in reaching the center from their residence, as highlighted by the following excerpts:

*P9: “Yes. Little waiting time is there, here there are only two chairs for treatment no and it takes some time to treat each patient too. This is the problem for waiting time, because only two chairs are there, if you increase that facility then it is good for others and for us too."*

*P6: “Hmm. I have a small baby, so waiting was a little difficult.”*

*P9: “We have to come in bus. That is a little problem. If we miss the bus then we have to come by rickshaw.”*

However, some participants also acknowledged that similar waiting periods are experienced in other dental clinics, making it more acceptable, as depicted in the following excerpt:

*P7: “Where ever we go, we need to wait. Private clinic or here, waiting will be compulsory (laughing). Who comes first will get treatment first and if we are late then our treatment also will be later.”*

### Post-visit perspectives

All participants recommended the center to others, with two participants (P2 and P5) specifically highlighting the free treatment offered to patients, as exemplified in the excerpts:

*P2: “I have told everyone about you all. That for free you remove teeth and give set.”*

*P5: “I have already told so many people. I have a lady near my house who needs upper teeth and they are seeing proposal for their daughter. She wants nice teeth, I told they remove teeth here for free and they also keep two or four teeth if it is missing also.”*

All participants revisited the center as they were provided with appointments, and among them, two participants also mentioned that they were willing to revisit out of self-interest.

*P4: “No they only gave appointment and I come at that time I come…”*

In the post-visit perspectives, a significant portion consisted of recommendations for enhancing dental care at the PHC. These suggestions encompassed various aspects, including the provision of free services, cost reduction, the addition of more chairs and staff to minimize waiting times, and the importance of basic amenities such as drinking water and shaded waiting areas, as emphasized by the participants. The diversity of these suggestions is evident in the following excerpts:

*P8: “It is good. But crown also should be done here. We are working class people. We can’t pay lot of money for treatment. We came here because it is free treatment. We were told crown also will be done.”*

*P10: “To give service you can have two extra chairs. People won’t have to wait then.”*

*P4: “Yes, if there are more doctors to test and do then it will be faster.”*

*P9: “Yes. Little waiting time is there, here there are only two chairs for treatment no and it takes some time to treat each patient too. This is the problem for waiting time, because only two chairs are there, if you increase that facility then it is good for others and for us too. Then they should put sheet where we sit here to wait.”*

*P6: “Yes. There could be more doctors treating us patients here. There could be more (dental) chairs.”*

P5: *"That’s enough. Then you need to treat kids too."*

## Discussion

The present qualitative research was conducted to delve into patients’ perception regarding the comprehensive dental services offered at the PHC through in-depth interviews. The findings illuminated that the prevailing outlook on care at the PHC was predominantly positive, indicating a favorable perception among the participants.

In this study, the most common reason for visiting the center was to obtain dentures, indicating a significant interest among in addressing their missing teeth. Following this, endodontic treatment ranked as the second most common reason. This is in contrast with a study conducted in Switzerland, where endodontic treatment was the most sought-after treatment, followed by dentures. ^30^ Some participants also mentioned that they visited because it offered free dental treatment. This suggests that the provision of free treatment increases the likelihood of patients utilizing the facility, aligning with findings of a Kerosuo et al., which notes that the treatment rate is influenced by the availability of free treatment. ^31^ When inquired about their previous source of dental care, a majority of participants had previously sought oral health care at private dental clinics. The transition to the center for subsequent treatment might be attributed to the availability of free treatment, which, in comparison to the costs incurred in private clinics, is likely to be considerably lower.

Most participants did not encounter any issues concerning the treatment provided, waiting times, or the availability of appointments. However, among those who faced difficulties, waiting time emerged as the primary concern. This aspect could have influenced their perceptions of the center and its facilities, aligning with findings from a study conducted by Inglehart et al. ^32^

Most of the participants in this study expressed satisfaction with the care they received at the center. Additionally, participants who visited the center primarily to alleviate pain reported relief. A study by Shrestha et al. indicated that patient satisfaction hinged on meeting the expectations and needs of the population. ^11^ Similarly, another study focusing on children revealed an overall improvement in the oral and general health of children who received comprehensive care, likely contributing to their satisfaction with dental treatment. ^13^ Similar results were observed in the present study with most participants expressing a positive opinion regarding the comprehensive care provided. This positive sentiment suggests that a holistic approach likely contributed to overall patient satisfaction by addressing the various facets of oral health and potentially related concerns.

The affordability of comprehensive care is a significant factor, as evidenced by a study conducted by Cruz et al, indicating that co-located clinics can enhance the cost-effectiveness of care. In line with this, a participant in our study highlighted the absence of a cost burden, emphasizing the convenience of receiving all necessary care in a single center and avoiding the expenses associated with traveling to different places for different treatments. ^18^ This can be appreciated in the following excerpt:

*P8: “One is to give crowns. Then, good if all treatments are provided here. We can come to one place. No need to go once here (center) and there (college). Good if everything is available in one place.”*

Regarding recommending the center to others, all participants in this study expressed their willingness to do so, with the majority having already recommended the center to others. The reasons cited for willingness to recommend included the availability of free treatment, quality of care and the convenience of accessing all care in one location. Notably, studies on orthodontic treatment, ^33^ fixed dental treatment ^34^ and aesthetic crown lengthening ^35^ have reported similar outcomes, where a substantial number of participants were willing to recommend the respective treatment to others. These findings were consistently attributed to the high levels of satisfaction with the treatments received.

When questioned about potential enhancements to the center for improved care, the participants predominantly expressed the need for increased resources. This included a demand for more dentists, additional dental chairs for treatment, a shaded waiting area for patients, and provision of drinking water in the waiting area. Participants specifically emphasized the requirement for more dentists and dental chairs to alleviate waiting times, aligning with the findings in a study conducted by Nair et al. ^20^ Additionally, participants voiced a desire for free dental crowns or fixed prosthetics at a lower cost, highlighting a perceived cost barrier to accessing paid dental services. This echoes the sentiments of the same study, where participants cited the high cost as a significant challenge, leading to difficult decisions on prioritizing family members for dental care when multiple individuals required treatment. ^20^ These results highlight the complex interplay of factors that affect the accessibility and outcomes of comprehensive dental care services.

### Limitations of the study

The scope of this study was confined to a single center and judgement sampling was used to enable the maximum variation in the sample, which might have introduced biases due to subjectivity. Therefore to enhance the generalizability of the findings, future investigations should consider these factors and incorporate multiple centers to increase the transferability of the outcomes.

## Conclusion

The overall results of this study revealed a predominantly positive outlook among the participants regarding the comprehensive dental services at the PHC. Through insightful interviews, both strengths and weaknesses of the center, as well as the oral health care provided, emerged. Although the majority of patients expressed satisfaction, a subset of participants offered constructive suggestions for improvement. The various suggestions, based on real experiences, offer potential for improvement, and can enhance the use of dental services, establishing an environment that fosters continuous enhancement and prioritizes patient-centric oral healthcare.

## Acknowledgement

We thank all the staff working at the Primary Health Centre for their co-operation towards completion of this research.

## Data Availability

A qualitative exploration of patients’ perception regarding the comprehensive dental services availed at a primary health center. figshare. Dataset. https://doi.org/10.6084/m9.figshare.24982380.v2. ^26^ Data are available under the terms of the Creative Commons Zero “No rights reserved” data waiver (CC0 1.0 Public domain dedication). The materials used in this study (questionnaire, participant information sheet, interview guide and example consent form) as extended data, uploaded to the repository alongside the underlying data. https://doi.org/10.6084/m9.figshare.24982380.v2. ^26^ Figshare: COREQ checklist for “A qualitative exploration of patients’ perception regarding the comprehensive dental services availed at a primary health center”. https://doi.org/10.6084/m9.figshare.24982380.v2. ^26^
