# Supplementary material for: A qualitative exploration of patients’ perception regarding the comprehensive dental services availed at a primary health center
Source: F1000Res. 2024 May 28;13:157. Originally published 2024 Mar 1. [Version 2] doi: 10.12688/f1000research.146781.2 (PMC11148531; doi:10.12688/f1000research.146781.2)
Supplement: Peer review history

# Reviewer report: Reviewer response for version 2

**DOI:** 10.5256/f1000research.166960.r283760

**Published:** 2024-06-03

**Authors:** Vinayak Kamath

**Affiliations:** 1 Goa Dental College and Hospital, Goa, India

I would like to thank the authors for the revision made to the manuscript.  I am fully satisfied with the changes made and have no further comments.

Is the work clearly and accurately presented and does it cite the current literature?

Yes

If applicable, is the statistical analysis and its interpretation appropriate?

I cannot comment. A qualified statistician is required.

Are all the source data underlying the results available to ensure full reproducibility?

Yes

Is the study design appropriate and is the work technically sound?

Yes

Are the conclusions drawn adequately supported by the results?

Yes

Are sufficient details of methods and analysis provided to allow replication by others?

Partly

Reviewer Expertise:

Oral Health

I confirm that I have read this submission and believe that I have an appropriate level of expertise to confirm that it is of an acceptable scientific standard.

# Reviewer report: Reviewer response for version 1

**DOI:** 10.5256/f1000research.160899.r259679

**Published:** 2024-05-22

**Authors:** Mallikarjun Sajjanshetty

**Affiliations:** 1 Balaji Dental College, Hyderabad, India

Well constructed manuscript wherein we get an insight into what are the patient perceptions in a dental setting where services are provided for free through a PPP mode. Interestingly conclusions provide a clue to other institutions / tertiary dental care centers to indulge into these activities to do their bit for the mankind.

 Of course perceptions/excerpts recorded are positive as the service provided is free of cost / relatively at a very low cost. This practice needs to be implemented every where.

 Work in the manuscript has been clearly presented and it is absolutely replicable. Study design is appropriate and conclusions drawn are in accordance to the data collected.

Is the work clearly and accurately presented and does it cite the current literature?

Yes

If applicable, is the statistical analysis and its interpretation appropriate?

Not applicable

Are all the source data underlying the results available to ensure full reproducibility?

Yes

Is the study design appropriate and is the work technically sound?

Yes

Are the conclusions drawn adequately supported by the results?

Yes

Are sufficient details of methods and analysis provided to allow replication by others?

Yes

Reviewer Expertise:

Health care research, Dental Caries, Tobacco control, Periodontal disease

I confirm that I have read this submission and believe that I have an appropriate level of expertise to confirm that it is of an acceptable scientific standard.

# Reviewer report: Reviewer response for version 1

**DOI:** 10.5256/f1000research.160899.r259671

**Published:** 2024-05-20

**Authors:** Vinayak Kamath

**Affiliations:** 1 Goa Dental College and Hospital, Goa, India

The authors have done an excellent job in exploring an interesting and important topic that may have significant impact on the quality of services and bridge the gap in access to oral health care.

 I am pleased to approve the manuscript.

 There are a few comments, which the authors need to address. They are as follows.

1. Introduction

- The authors may emphasize why this specific research is critical by linking it directly to gaps in existing literature or practical needs, to strengthen the argument for the study's relevance.
- Briefly mention the value of qualitative research in achieving the study objectives, which would justify the methodology used.

1. Methods

- The use of judgment sampling is appropriate but briefly discuss the potential biases and how they are mitigated.
- A single interviewer who regularly attended the PHC has conducted the interview. What steps were taken to reduce interviewer’s bias? Has this familiarity influenced participant’s responses due to social desirability bias?
- To assess content validity, Was CVI/CVR used?
- The authors have estimated a sample size of 25. More details may be provided regarding this for the benefit of readers.
- The researchers may provide cumulative frequency graph supporting their judgment that saturation was achieved as supplementary data.
- Translating interviews from the local language to English and transcribing them verbatim could introduce errors or loss of nuanced meaning. The translation process needs to be presented thoroughly.
- Any reason why the study did not include repeat interviews, which could have provided additional depth and validation of initial findings
- More detailed steps on the data analysis process would enhance transparency.(Eg As described by Braun and Clarke)

1. Discussion & Conclusion–

- While the study’s scope limitation is mentioned, discussing other potential limitations and how they might affect the findings would add rigor.Eg:

1. Interview times range from 5 to 14 mins which may be relatively short in depth qualitative interview limiting the depth of data collected.
2. Only basic demographic details (age, gender, area of residence) were collected. More detailed demographic information could provide a richer context for the findings.

- Based on the findings of the study, would the authors like to provide more specific recommendation for future research and for policymakers or practitioners?

Is the work clearly and accurately presented and does it cite the current literature?

Yes

If applicable, is the statistical analysis and its interpretation appropriate?

I cannot comment. A qualified statistician is required.

Are all the source data underlying the results available to ensure full reproducibility?

Yes

Is the study design appropriate and is the work technically sound?

Yes

Are the conclusions drawn adequately supported by the results?

Yes

Are sufficient details of methods and analysis provided to allow replication by others?

Partly

Reviewer Expertise:

Oral Health

I confirm that I have read this submission and believe that I have an appropriate level of expertise to confirm that it is of an acceptable scientific standard, however I have significant reservations, as outlined above.
